# Health state utilities for metastatic breast cancer

**DOI:** 10.1038/sj.bjc.6603326

**Published:** 2006-09-12

**Authors:** A Lloyd, B Nafees, J Narewska, S Dewilde, J Watkins

**Affiliations:** 1 United BioSource Corporation, 20 Bloomsbury Square, London WC1A 2NS, UK; 2 Eli Lilly and Company Limited, Priestley Road, Basingstoke, Hampshire RG24 9NL, UK

**Keywords:** breast cancer, utility, quality of life, standard gamble

## Abstract

The aim of the study was to obtain United Kingdom-based societal preferences for distinct stages of metastatic breast cancer (MBC) and six common toxicities. Health states were developed based on literature review, iterative cycles of interviews and a focus group with clinical experts. They described the burden of progressive, responding and stable disease on treatment; and also febrile neutropenia, stomatitis; diarrhoea/vomiting; fatigue; hand-foot syndrome (grade 3/4 toxicities) and hair loss. One hundred members of the general public rated them using standard gamble to determine health state utility. Data were analysed with a mixed model analysis. The study sample was a good match to the general public of England and Wales by demographics and current quality of life. Stable disease on treatment had a utility value of 0.72, with a corresponding gain of +0.07 following a treatment response and a decline by 0.27 for disease progression. Toxicities lead to declines in utility between 0.10 (diarrhoea/vomiting) and 0.15 (febrile neutropenia). This study underlines the value that society place on the avoidance of disease progression and severe side effects in MBC. This may be the largest preference study in breast cancer designed to survey a representative general public sample.

Incidence rates of breast cancer have continued an upward trend in recent years, increasing by 70% since 1971 and by 15% in the 10 years to 2000. In 2002, approximately 11 500 women died from breast cancer in England and Wales (Office for National Statistics, 2003).

Recurrence of breast cancer and the diagnosis of metastatic breast cancer (MBC) can be particularly devastating for women and their families. Patients' health-related quality of life (HRQL) can be impaired by the experience of symptoms from the metastatic disease, the burden of the treatment and the individual's ability to cope (McCorkle, 1973). Decreased functioning necessitates alterations in day-to-day activities, personal relationships, care-giving demands and psychosocial concerns (Lam and Fielding, 2003).

Robust, well-designed and validated disease-specific measures of HRQL exist to measure the impact of disease and treatment in breast cancer. However, determining the effectiveness of a treatment within a cost-utility (cost per quality-adjusted life year or QALY) framework requires the collection of preference- (or utility-) based HRQL data. One means of doing this is by using the EuroQol Group (EQ-5D) (Rabin and de Charro, 2001), a generic preference-weighted HRQL measure that produces an individual utility score. Patients complete a simple descriptive system, characterising them into one of 243 possible health states that have previously been valued by members of the general public. Generic HRQL instruments may lack the sensitivity of disease-specific measures, and the generalisability of data collected within a clinical trial can be limited by the study entry criteria, and drop-out from the trial that may occur differentially for patients with severely compromised health or HRQL. If utility values for specific subgroups or adverse events are required, these data could be limited if the number of people experiencing such events is low.

An alternative to this approach is to generate detailed health state descriptions of health states related to MBC. Utility scores for these health states can be determined by eliciting preferences from members of the general public or patients. Preferences for health states can be estimated by eliciting the maximum level of risk (of being dead) that the individual is willing to accept in order to avoid each health state (referred to as standard gamble (SG) (Torrance, 1986; Bennett and Torrance, 1996).

Some reported studies have captured utility data for MBC (Earle *et al*, 2000; Perez *et al*, 2001; Franic and Pathak, 2003; Franic *et al*, 2005). However, these studies are generally based on the preferences of patients and/or doctors and do not reflect societal preferences. There are fewer reports that focus on different stages specifically within MBC or specific adverse events related to chemotherapy regimens.

This study was designed to elicit societal preferences for health states describing different MBC disease states (stable on treatment, responding disease and progressive disease). In addition, these disease states were combined with five different grade 3/4 toxicities and hair loss.

## MATERIALS AND METHODS

### Development of health states

The development of the health states was based on information from different sources. This included a rapid literature review, exploratory interviews with expert physicians, one focus group with oncology specialist nurses and further content validation interviews. The health states were produced for a societal valuation study, which would include both women and men. Therefore, they were designed to be easily understandable, and gender neutral as far as was possible. The health states also made no explicit reference to cancer. The health states were designed to describe a 3-week period. They were designed for use in an economic model where patients will cycle between health states, with transition probabilities determined from clinical trials.

### Rapid literature review

A rapid literature review was conducted, primarily designed to identify the qualitative nature of the HRQL burden in MBC. Existing patient preference studies or qualitative studies in this area were given special attention. We were less interested in studies that simply reported data from the use of HRQL questionnaires. Several studies described HRQL in patients with MBC (Sprangers *et al*, 1996; Brady *et al*, 1997; Geels *et al*, 2000; Perez *et al*, 2001; O'Shaughnessy *et al*, 2002; Chung and Carlson, 2003; Hopwood *et al*, 2004; Luoma and Hakamies-Blomqvist, 2004; Jones *et al*, 2005).

Seven main areas of HRQL and disease burden in MBC were identified. These included physical, social, sexual, cognitive and emotional functioning, and toxicities and symptoms (Luoma and Hakamies-Blomqvist, 2004). The review also highlighted how frequently disease-specific measures, such as the FACT-B (Brady *et al*, 1997) and European Organisation for Research and Treatment of Cancer Core Quality of Life questionnaire-BR23 (Sprangers *et al*, 1996), had been used. The content of these measures provided useful descriptive information regarding ways in which women are affected by breast cancer.

The six toxicities included in the health states were febrile neutropenia, hand-foot syndrome, stomatitis, fatigue, diarrhoea and vomiting (all grade 3/4), and hair loss. Hair loss was included because it was rated as being a key concern to women receiving chemotherapy treatment (Hopwood *et al*, 2004). The toxicities were selected because they occurred in over 6% of patients in data from two recent clinical trials in MBC with different therapies (capecitabine, docetaxel and paclitaxel) (O'Shaughnessy *et al*, 2002; Jones *et al*, 2005).

### Exploratory interviews

An interview discussion guide was developed to understand the nature of stable, responding and progressive MBC in terms of the symptoms and impact on HRQL. The guide queried experts about the symptom burden that patients' experience (especially pain and fatigue), the nature and burden of the toxicities and areas of functioning (social, sexual, cognitive, physical and emotional). For each area, specific concerns were noted. For example in emotional functioning, feelings of worry, hopelessness, anxiety and depression were explored. Experts were asked to describe patient burden in three base disease health states – stable on treatment, responding disease (50% reduction in patient's five largest tumours) and progressive disease (25% growth in patient's five largest tumours).

Consultant or specialist registrar grade oncologists (*n*=7) were identified via an online database of UK-based medical specialists and recruited to take part in the interviews. Telephone interviews were conducted by trained interviewers, recorded and transcribed by a professional transcription agency.

Clinicians described how pain, fatigue, hair loss and diarrhoea/vomiting markedly affected HRQL in these patients. The impact of MBC on physical functioning was addressed using four domains: self-care, caring for your environment, shopping/outdoor duties and ability to work. Clinicians reported that physical functioning varies across different states of tumour response. Those with responding disease would be able to manage most tasks (except perhaps the ability to work). Those with stable disease would be likely to be able to care for themselves, but perhaps not do duties outside the home and would probably have given up working. Patients with progressive disease would manage some self-care, but otherwise would be unable to function at the other levels without assistance. A good consensus emerged from the clinicians regarding the impact of MBC on sexual and social functioning.

The severity and impact of treatment-related toxicities was also described and clinicians reported that they generally did not vary across disease state. Patients with responding disease were considered better able to cope with side effects than patients with stable disease. The impact of symptoms is likely to be greater for patients with progressive disease as they are less able to cope.

### Health states: first draft

The first draft of the health states were produced based upon the interviews and literature review. Fifteen health states were developed to reflect accurately the input from the clinicians. The health states did not make explicit reference to cancer because it was felt that this may have a significant biasing effect on how people value them. In the main study, the interviewers believed that some participants correctly guessed that the states described cancer, but nobody indicated that they recognised them as breast cancer. Interviewers did not confirm whether they described cancer or not.

### Health states validation: focus group

A focus group led by a trained facilitator (BN) further explored the validity of the health states. The health states were reviewed for appropriateness and accuracy by four oncology specialist nurses. Changes were made to the states based upon this feedback, much of it focused on better describing the severity of different aspects. The nurses concluded that the health states were slightly limited in their ability to capture the full range of patients' experience, but were reasonably accurate descriptions. Some suggested changes relating to toxicities and symptoms were incorporated.

### Health states validation: interviews

Three clinical oncologists were asked to review the health states for accuracy during an in-depth telephone de-briefing interview. Qualitative and quantitative differences in HRQL and symptom burden between varying stages and chemotherapy-related toxicities were examined. Each health state was queried for the accuracy of each part of the description.

Transcripts from the interviews were independently analysed by three researchers. The oncologists generally agreed that the health descriptions were an accurate reflection of the states. A few minor suggestions were made, relating to the wording.

A final review of the health states was undertaken by two psychometric experts with expertise in developing measures of HRQL and health states. Their comments and suggestions, which did not contradict those of the clinical reviewers, were incorporated into the health state descriptions at this stage. An example of one of the health states is included here (Box 1).

### Pilot study

The health states were piloted with five members of the general public in a conventional SG interview. Following the completion of the interview, participants were de-briefed to identify any problems in the content of the health states or comprehensibility and language issues. No issues emerged from the pilot study and no revisions were needed.

### Main study

Members of the public provided estimates of utility for the MBC-related health states in line with the recommendations of National Institute for Health and Clinical Excellence (NICE, 2004) and the Washington Panel on Cost Effectiveness (Gold *et al*, 1996). Participants were recruited from Greater London through advertisements in local newspapers and from an existing UBC database of willing survey participants. Interviews were conducted by trained interviewers at the UBC offices. The purpose of the interview was fully explained to participants at the start. All participants provided written informed consent and completed a sociodemographic questionnaire. Participants also completed the EQ-5D to rate their current health.

Health states describing *full health, dead, own current health* and *worst health* (designed to be worse than any of the other MBC health states) were used in addition to the health states that were developed from the interviews. The SG interview included two tasks. visual analogue scale (VAS) and SG utility methods (Torrance, 1986; Bennett and Torrance, 1996) were used to elicit participants' utilities for the health states. Each health state description (including ‘dead’) was first rated on a VAS referred to as the ‘feeling thermometer’. This had a lowest value of zero and a highest value of 100 (anchored by the ‘full health’ card).

In the SG task for each health state, patients were asked to choose one of three options: (1) to live in the hypothetical health state with certainty for the next 10 years; (2) to choose between various probabilities of having either *full health* or *worst health* for the next 10 years; or (3) to indicate that the two previous options were equal. Probabilities for option 2 (full health and worst health) were varied sequentially until the patient was indifferent between them. Finally, the worst health state was assessed, based on a gamble between *full health* and *dead*. If any health states had been rated as worse than *dead* in the VAS task, then participants were asked to choose between the certain prospect of *dead* and a gamble between the health state in question and *full health*. The utility value for the *worst health* state was determined against *dead* and this was used to recalibrate the values for the other health states on the dead (0) to full health (1) scale.

### Data collection

The study included 18 health states (15 health states developed, one own health, one full health and one dead state) (Appendix A). These were considered too many health states for each participant to review so participants completed some anchor states (stable and responding disease with no side effects, and progressive disease) and half of the remaining states (by reviewing one combination of disease state and toxicity which made 10 in total). Equal proportions of men and women were recruited.

### Statistical analysis

The study was designed to collect data from 100 people. This was not determined by a formal power analysis partly because there was no specific hypothesis to test. The demographic and EQ-5D data were summarised and compared to the UK population. The demographic profile of the study participants was compared to the 2001 national census data for England and Wales (Office of National Statistics, 2001). In addition the EQ-5D data were summarised to determine how closely the sample matched a previous national survey of health in terms of health status and HRQL. Percentages of the sample reporting moderate or extreme problems on each dimension of the EQ-5D were compared to results of a UK National Survey (Kind *et al*, 1998).

The health state valuations from the SG interview were analysed using a mixed model analysis with random effects on the patient level to determine the change in utility score associated with moving between stages of disease and from no toxicity to one of the toxicities included. The raw data were transformed using a logistic transformation (transformed utility=log ((1-utility)/utility)). Negative utility scores were changed to a positive utility value of 0.02, which very slightly increased the mean utility value and reduced the variation. This was done in order to obtain a normal distribution suitable to be used in a standard regression model. Age would be expected *a priori* to affect preferences and so it was also included in the analysis. Older participants have more experience of disease but also have fewer years of life left on average both of which may affect their preferences around risk.

A further analysis was also conducted to explore the effect of gender on participants' preferences. It was hypothesised that if participants recognised the health state descriptions as being related to breast cancer then it is possible that men would view the states differently to women. Therefore the same mixed model analysis was undertaken with gender included as a dummy variable. Different model specifications were compared and, using the Likelihood Ratio test, a model that included gender and the interaction between gender and disease progression was settled on as the most parsimonious.

## RESULTS

### Participant characteristics

Of 106 respondents, 100 completed the full interview (data collection continued until 100 datasets were available). Six participants were not included in the final analysis because in the interviewer's opinion they failed to understand the SG task. The demographic profile of the participants was similar to the UK population data (Table 1). There was a higher proportion of people from ethnic minorities (28%) in our sample and quite a high proportion with university education (38%). Apart from these differences the study sample was a fair representation of the general public in England and Wales. The sample also had a similar distribution of HRQL impairments to the national sample reported by Kind *et al* (1998). The participants in the present study were less likely to report severe problems in all the EQ-5D dimensions. No participants reported extreme problems with mobility, self-care or pain/discomfort.

The mean EQ-5D VAS score for own current health was 82.49 (s.d.=16.19) and the mean EQ-5D single index score was 0.922 (s.d.=0.172). Mean VAS score for own health from the SG interview in the study sample was 87.2 (s.d.=13.86). Mean SG utility score for current health was 0.90 (s.d.=0.16).

### Health state utility values for MBC

The mixed model analysis revealed that all disease states and toxicities were independently significant predictors of utility (Table 2). The base state (stable disease with no side effects) had a utility value of 0.715. Moving to responding disease from stable on treatment produced a significant utility gain (+0.075). Moving from stable disease to progressive disease led to a significant decrement in utility (−0.272). The toxicities were all associated with a significant decrement in utility compared to no toxicity. Using the results of the mixed model it is possible to estimate a utility value for any combination of disease states and toxicities. This is done by adding the parameter estimates from the mixed and back-transforming them using 
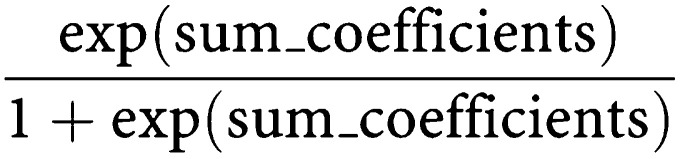


For example, a 40-year old patient who is stable on treatment, with diarrhoea and vomiting, hair loss and fatigue has a utility value of: 
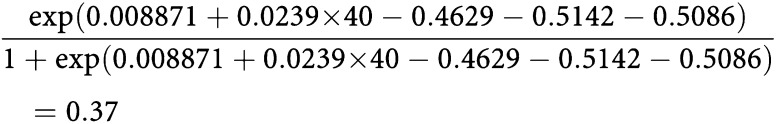


### Age

There was a significant association between study participants' age and utility values from the mixed model (*P*=0.0006). Stable disease with no side effects was given a utility value of 0.72 by a person of 40; while a person aged 50 rated the same health state as 0.77. The utility values presented in Table 3 have been estimated based upon the preferences of someone aged 38.2 to match the UK census data (Table 1).

### Gender

In the mixed model the gender by progression interaction was significant (parameter estimate: 0.3973, *P*=0.0202) but the gender term was not (0.09653, *P*=0.743) indicating that male participants placed a greater disutility on disease progression compared to women. The disutility associated with disease progression was equal to −0.220 for women (stable disease=0.725 and disease progression=0.505) but equalled −0.322 for men (stable disease=0.705 and disease progression=0.384). Men and women rated stable disease similarly, and there were no differences in their interpretation of the toxicities. Note that the disutility changes with the presence of side effects, because the logistic transformation is not linear.

## DISCUSSION

This study reports health state utility values from the UK general public for health states related to stable, responding and progressive MBC, and six toxicities related to chemotherapy treatment. Health state descriptions were developed from interviews and focus groups with experts in breast cancer, reviewed by clinical and psychometric experts and piloted on members of the general public.

Data from the study show the significance that the general public place on the changes in HRQL seen in MBC. Disease progression has the largest impact on HRQL, being associated with a mean utility decrement of 0.272. However, the data also underline how important the avoidance of chemotherapy-related side effects is. Each of the toxicities led to a decline in utility of at least 0.103. The study revealed that hair loss is given similar importance, in terms of utility loss, as grade 3/4 side effects such as fatigue and hand-foot syndrome. Participants considered febrile neutropenia and stomatitis to be the worst toxicities.

There were also gender differences in the value or disutility that participants placed on disease progression. Male participants indicated that they thought there was a greater decline in utility associated with disease progression compared to female participants. The reason for this remains unclear but it may be that female participants considered family responsibilities and child care more in rating health states and so were less willing to accept risks to avoid progression.

The analysis revealed that the age of the study participants had a positive correlation with utility values. Younger participants considered the health states to be worse than older participants. This may be because older participants are more risk averse and less willing to risk their health in the remaining years of life. It is also possible that older participants give higher ratings to the health states because they perceived them as less of a departure from their current health state.

This study employed an efficient method of obtaining utility values for different disease states and toxicities. This allowed much greater flexibility in estimating utility values for different combinations of toxicities and disease states. This arguably allows for the development of more clinically meaningful analyses of the impact of treatments. The approach could also be used to capture different levels of severity, approaches to therapy and provisions of palliative care. The use of the mixed-model analysis, combined with efficient designs means that study participants only have to assess a minimum number of health states and yet it is possible to estimate utility scores for a large number of combinations.

The present sample was a reasonably good match to the UK population. Ethnic minorities were over-represented, perhaps reflecting the population of London rather than the whole of England and Wales. The present study sample reported lower frequencies of moderate and/or severe problems on the mobility, self-care and pain/discomfort dimensions of the EQ-5D (but not usual activities or anxiety/depression where the distribution of extreme problems was similar to the results from Kind *et al*, 1998). Our participants were required to attend for interview and so needed to be reasonably mobile, which may explain why fewer people reported some extreme problems. The study by Kind and co-workers used a postal survey to collect data, and so people with poorer levels of mobility could participate more easily.

The study has some significant limitations that deserve to be highlighted. The health states were developed from a literature review and interviews with clinicians with no direct input from patients to validate them. Validating the health states would require a review from people who have recently experienced each of the toxicities. It would also require a content validation with people who are currently receiving treatment for MBC. The present method used to develop health states is a simple pragmatic approach. We have tried to improve the validity of the health states through rounds of interviews and a focus group, including different types of clinical staff in order to arrive at a consensus. The use of clinicians also means that we are able to get a broad view of the impact of MBC based on experience with a large number of patients. This helps us to avoid the health states reflecting idiosyncratic variation, which can be very substantial in metastatic disease.

The data from this study are not easy to compare to other published studies for a number of reasons. A utility value for febrile neutropenia in MBC without hospitalisation of 0.66 has been reported, which is slightly higher than the equivalent value of 0.565 in the present study (Launois *et al*, 1996). This difference is partly due to the difference in hospitalisation. In addition, values for stable MBC with toxicities ranging from 0.50 to 0.80 have been reported, matching the data from the present study (Launois *et al*, 1996; Hillner *et al*, 1992). However, both these studies elicited nurses rather than societal preferences and so it is not easy to compare them. In a major review of utility scores in oncology, Earle *et al* (2000) did not identify any societal-based valuations of breast cancer health states. Our searches have also failed to find any other studies that report societal valuations in breast cancer. Other studies in breast cancer with patients and nurses may also be difficult to compare because different preference elicitation methods such as time trade off or rating scales have been used.

This study reports what is probably the largest representative sample of residents of England and Wales to have stated their preferences for health states related to MBC. This study may also be used as a basis for further studies to obtain utility data with larger samples across UK and also possibly with patients to determine the weight they place on different outcomes. The methodology has allowed us to estimate the utility of a very large number of combinations of side effects and disease states within MBC.

## Figures and Tables

**Box 1:**
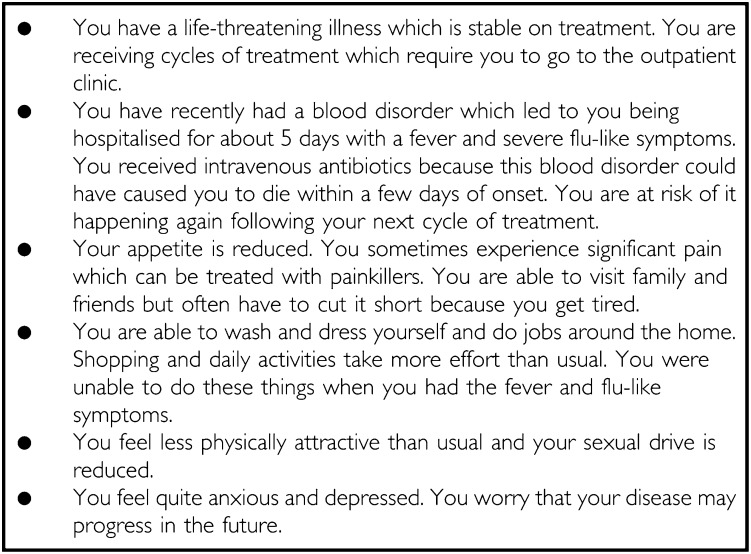
The health state designed to describe a patient with metastatic breast cancer who is stable on treatment but with grade 3/4 febrile neutropenia

**Table 1:** Demographic profile of study sample suggest one decimal place for sample results

	**Study sample (*N*=100)**	**UK census and ONS data 2001–2004**
Age mean (s.d.)	40.16 (13.59)	38.2
Gender (*N*/Tot, %female	50% Female	51% Female
		
*Ethnic group*		
White	72%	92.1%
Black	8%	2.0%
Asian	11%	4.0%
Other (includes mixed race, Jewish, Irish)	9%	1.9%
		
*Employment status*		
Full time	48%	—
Part time	19%	—
Home maker	2%	4.6%
Disabled	5%	4.2%
Retired	6%	9.8%
Student	11%	1.9%
Other	9%	2.3%
		
*Education – leaving age*		
No formal qualifications	3%	—
GCSE/O levels (16 years)	16%	—
A levels (18 years)	15%	—
Vocational or work based	10%	—
University degree	38%	—
Other	18%	—

GCSE=General Certificate of Secondary Education; ONS=UK Office of National Statistics.

**Table 2:** Results of the mixed model analysis

**Parameter**	**Parameter estimate**	**s.e.**	**d.f.**	***t*-value**	***P*-value**
Intercept	0.008871	0.3196	97	0.03	0.9779
Age	0.0239	0.006946	862	3.44	0.0006
Treatment response	0.4063	0.05521	862	7.36	<0.0001
Disease progression	−1.1477	0.1031	862	−11.14	<0.0001
Febrile neutropenia	−0.6603	0.08501	862	−7.77	<0.0001
Diarrhoea and vomiting	−0.4629	0.09929	862	−4.66	<0.0001
Hand-foot syndrome	−0.5184	0.09929	862	−5.22	<0.0001
Stomatitis	−0.6634	0.09929	862	−6.68	<0.0001
Fatigue	−0.5142	0.09929	862	−5.18	<0.0001
Hair loss	−0.5086	0.09929	862	−5.12	<0.0001

d.f.=degrees of freedom; s.e.=standard error.

The analysis included 969 observations.

**Table 3:** Utility value of base state (stable MBC on treatment with no toxicity) and utility gains and decrements associated with departures from this health state

**Parameter**	**Utility values**
Base state – stable disease with no toxicity	0.715
Treatment response	+0.075
Disease progression	−0.272
Febrile neutropenia	−0.150
Diarrhoea and vomiting	−0.103
Hand-foot syndrome	−0.116
Stomatitis	−0.151
Fatigue	−0.115
Hair loss	−0.114

MBC=metastatic breast cancer.

## **Appendix A**

### HEALTH STATES

#### Stable MBC with no side effects (please note that the health states used in the interviews did not include these titles)

- You have a life-threatening illness which is stable on treatment. You are receiving cycles of treatment which require you to go to the outpatient clinic.
- Your appetite is reduced. You sometimes experience significant pain which can be treated with painkillers. You are able to visit family and friends but often have to cut it short because you get tired.
- You are able to wash and dress yourself and do jobs around the home. Shopping and daily activities take more effort than usual.
- You feel less physically attractive than usual and your sexual drive is reduced.
- You feel quite anxious and depressed. You worry that your disease may progress in the future.

#### Stable MBC with febrile neutropenia

- You have a life-threatening illness which is stable on treatment. You are receiving cycles of treatment which require you to go to the outpatient clinic.
- You have recently had a blood disorder which led to you being hospitalised for about 5 days with a fever and severe flu-like symptoms. You received intravenous antibiotics because this blood disorder could have caused you to die within a few days of onset. You are at risk of it happening again following your next cycle of treatment.
- Your appetite is reduced. You sometimes experience significant pain which can be treated with painkillers. You are able to visit family and friends but often have to cut it short because you get tired.
- You are able to wash and dress yourself and do jobs around the home. Shopping and daily activities take more effort than usual. You were unable to do these things when you had the fever and flu-like symptoms.
- You feel less physically attractive than usual and your sexual drive is reduced.
- You feel quite anxious and depressed. You worry that your disease may progress in the future.

#### Stable MBC with fatigue

- You have a life-threatening illness which is stable on treatment. You are receiving cycles of treatment which require you to go to the outpatient clinic.
- You often feel extremely tired and weak all over. Your tiredness is not relieved by rest. Most of the time you are frustrated by being too tired to do the things you used to do easily.
- Your appetite is reduced. You sometimes experience significant pain which can be treated with painkillers. You are able to visit family and friends but often have to cut it short because you get tired.
- You are able to wash and dress yourself some of the time. You cannot do jobs around the home, shopping or other daily activities because you are too tired.
- You feel less physically attractive than usual and your sexual drive is reduced.
- You feel quite anxious and depressed. You worry that your disease may progress in the future.

#### Stable MBC with stomatitis

- You have a life-threatening illness which is stable on treatment. You are receiving cycles of treatment which require you to go to the outpatient clinic.
- You have recently had very painful sores in your mouth which meant that you could not eat or drink properly. The pain from your mouth disturbed your sleep. You were taking pain medication and mouthwash to treat it. This lasted for about 7 days. You are at risk of it happening again following your next cycle of treatment.
- Your appetite is reduced. You sometimes experience significant pain which can be treated with painkillers. You are able to visit family and friends but often have to cut it short because you get tired.
- You are able to wash and dress yourself and do jobs around the home. Shopping and daily activities take more effort than usual. You were unable to do these things when you had the painful sores in your mouth.
- You feel less physically attractive than usual and your sexual drive is reduced.
- You feel quite anxious and depressed. You worry that your disease may progress in the future.

#### Stable MBC with hand-foot syndrome

- You have a life-threatening illness which is stable on treatment. You are receiving cycles of treatment which require you to go to the outpatient clinic.
- You have recently suffered from very painful sores on your hands and feet. Your feet were so badly blistered that you could not walk and you had great difficulty using your hands. This lasted for 3–4 weeks.
- Your appetite is reduced. You sometimes experience significant pain which can be treated with painkillers. You are able to visit family and friends but often have to cut it short because you get tired.
- You are able to wash and dress yourself and do jobs around the home. Shopping and daily activities take more effort than usual. You were unable to do these things when you had the sore hands and feet.
- You feel less physically attractive than usual and your sexual drive is reduced.
- You feel quite anxious and depressed. You worry that your disease may progress in the future.

#### Stable MBC with diarrhoea and vomiting

- You have a life-threatening illness which is stable on treatment. You are receiving cycles of treatment which require you to go to the outpatient clinic.
- You recently had a bout of sickness that lasted 2–3 days. You felt very sick and were vomiting occasionally. You also had frequent severe diarrhoea. You were taking medication to treat the diarrhoea and vomiting. You are at risk of it happening again following your next cycle of treatment.
- Your appetite is reduced. You sometimes experience significant pain which can be treated with painkillers. You are able to visit family and friends but often have to cut it short because you get tired.
- You are able to wash and dress yourself and do jobs around the home. Shopping and daily activities take more effort than usual. You were unable to do these things when you had the sickness and diarrhoea.
- You feel less physically attractive than usual and your sexual drive is reduced.
- You feel quite anxious and depressed. You worry that your disease may progress in the future.

#### Stable MBC with hair loss

- You have a life-threatening illness which is stable on treatment. You are receiving cycles of treatment which require you to go to the outpatient clinic.
- You have lost all of your hair.
- Your appetite is reduced. You sometimes experience significant pain which can be treated with painkillers. You are able to visit family and friends but often have to cut it short because you get tired.
- You are able to wash and dress yourself and do jobs around the home. Shopping and daily activities take more effort than usual.
- You feel less physically attractive than usual and your sexual drive is reduced.
- You feel quite anxious and depressed. You worry that your disease may progress in the future.

#### Responding MBC with no side effects

- You have a life-threatening illness which is responding to treatment. You are receiving cycles of treatment which require you to go to the outpatient clinic.
- Your appetite is returning. You sometimes experience pain which can be treated with painkillers. You are sometimes tired but are able to visit family and friends.
- You are able to wash and dress yourself and do jobs around the home. Shopping and daily activities can sometimes be tiring.
- You sometimes feel less physically attractive and your sexual drive is reduced.
- You worry about your condition but are somewhat hopeful about the future.

#### Responding MBC with febrile neutropenia

- You have a life-threatening illness which is responding to treatment. You are receiving cycles of treatment which require you to go to the outpatient clinic.
- You have recently had a blood disorder which led to you being hospitalised for about 5 days with a fever and severe flu like symptoms. You received intravenous antibiotics because this blood disorder could have caused you to die within a few days of onset. You are at risk of it happening again following your next cycle of treatment.
- Your appetite is returning. You sometimes experience pain which can be treated with painkillers. You are sometimes tired but are able to visit family and friends.
- You are able to wash and dress yourself and do jobs around the home. Shopping and daily activities can sometimes be tiring. You were unable to do these things when you had the fever and flu like symptoms.
- You sometimes feel less physically attractive and your sexual drive is reduced.
- You worry about your condition but are somewhat hopeful about the future.

#### Responding MBC with fatigue

- You have a life-threatening illness which is responding to treatment. You are receiving cycles of treatment which require you to go to the outpatient clinic.
- You often feel tired and weak all over. Most of the time you are frustrated by being too tired to do the things you used to do easily.
- Your appetite is returning. You sometimes experience pain which can be treated with painkillers. You sometimes do not have the energy to see family and friends.
- You are able to wash and dress yourself and do jobs around the home. Shopping and daily activities take some effort.
- You sometimes feel less physically attractive and your sexual drive is reduced.
- You worry about your condition but are somewhat hopeful about the future.

#### Responding MBC with stomatitis

- You have a life-threatening illness which is responding to treatment. You are receiving cycles of treatment which require you to go to the outpatient clinic.
- You have recently had very painful sores in your mouth which meant that you could not eat or drink properly. The pain from your mouth disturbed your sleep. You were given pain medication and mouthwash to treat it. This lasted for about 7 days. You are at risk of it happening again following your next cycle of treatment.
- Your appetite is returning. You sometimes experience pain which can be treated with painkillers. You sometimes do not have the energy to see family and friends.
- You are able to wash and dress yourself and do jobs around the home. Shopping and daily activities can sometimes be tiring. You were unable to do these things when you had the painful sores in your mouth.
- You sometimes feel less physically attractive and your sexual drive is reduced.
- You worry about your condition but are somewhat hopeful about the future.

#### Responding MBC with hand-foot syndrome

- You have a life-threatening illness which is responding to treatment. You are receiving cycles of treatment which require you to go to the outpatient clinic.
- You have recently suffered from very painful sores on your hands and feet. Your feet were so badly blistered that you could not walk and you had great difficulty using your hands. This lasted for 3–4 weeks.
- Your appetite is returning. You sometimes experience pain which can be treated with painkillers. You sometimes do not have the energy to see family and friends.
- You are able to wash and dress yourself and do jobs around the home. Shopping and daily activities can sometimes be tiring. You were unable to do these things when you had the sore hands and feet.
- You sometimes feel less physically attractive and your sexual drive is reduced.
- You worry about your condition but are somewhat hopeful about the future.

#### Responding MBC with diarrhoea and vomiting

- You have a life-threatening illness which is responding to treatment. You are receiving cycles of treatment which require you to go to the outpatient clinic.
- You recently had a bout of sickness that lasted 2–3 days. You felt very sick and were vomiting occasionally. You also had frequent severe diarrhoea. You were taking medication to treat the diarrhoea and vomiting. You are at risk of it happening again following your next cycle of treatment.
- Your appetite is returning. You sometimes experience pain which can be treated with painkillers. You sometimes do not have the energy to see family and friends.
- You are able to wash and dress yourself and do jobs around the home. Shopping and daily activities can sometimes be tiring. You were unable to do these things when you had the sickness and diarrhoea.
- You sometimes feel less physically attractive and your sexual drive is reduced.
- You worry about your condition but are somewhat hopeful about the future.

#### Responding MBC with hair loss

- You have a life-threatening illness which is responding to treatment. You are receiving cycles of treatment which require you to go to the outpatient clinic.
- You have lost all of your hair.
- Your appetite is returning. You sometimes experience pain which can be treated with painkillers. You sometimes do not have the energy to see family and friends.
- You are able to wash and dress yourself and do jobs around the home. Shopping and daily activities can sometimes be tiring.
- You sometimes feel less physically attractive and your sexual drive is reduced.
- You worry about your condition but are somewhat hopeful about the future.

#### Progressive MBC

- You have a life-threatening illness and your condition is getting worse. You are on stronger medication to relieve any increasing pain. You experience severe fatigue. You have lost your appetite and have experienced significant weight loss.
- You feel too tired to go out or to see family and friends. Some of your relationships with them are strained.
- You are able to wash and dress yourself with some assistance. You are often unable to do jobs around the house or other daily activities. You are reliant on others.
- You feel your physical appearance is deteriorating and you have little or no sexual drive.
- You feel depressed and feel dependent on your family and friends. You have little hope for the future.

#### Worst health

- Your condition is critical. You are receiving strong medication to relieve any increasing pain and nausea but it is not helping. You experience constant severe fatigue. You hardly eat and you have lost a large amount of weight.
- You are too tired to see family and friends and conversing with visitors has become difficult.
- You may have been moved to a palliative care unit or be receiving hospice service at home. You are confined to a bed or chair, and need assistance using the toilet, washing, dressing and eating, etc.
- Your gaunt, frail physical appearance is alarming to yourself and your family.
- You feel a burden on loved ones and your lack of independence is demoralising. You have lost hope for recovery. You are afraid of dying in pain and have begun discussing your concerns with your caregivers.

## References

[bib1] Bennett KJ, Torrance GW (1996) Measuring health state preferences and utilities: rating scale, time trade-off, and standard gamble techniques. In Quality of Life and Pharmacoeconomics in Clinical Trials, Spilker B (ed) pp 253–265. Philadelphia, PA: Lippincott-Raven Publishers

[bib2] Brady MJ, Cella DF, Mo F, Bonomi AE, Tulsky DS, Lloyd SR, Deasy S, Cobleigh M, Shimoto G (1997) Reliability and validity of the Functional Assessment of Cancer Therapy-Breast quality-of-life instrument. J Clin Oncol 15(3): 974–986 9060536 10.1200/JCO.1997.15.3.974

[bib3] Chung CT, Carlson RW (2003) Goals and objectives in the management of metastatic breast cancer. Oncologist 8(6): 514–520 14657529 10.1634/theoncologist.8-6-514

[bib4] Earle CC, Chapman RH, Baker CS, Bell CM, Stone PW, Sandberg EA, Neumann PJ (2000) Systematic overview of cost-utility assessments in oncology. J Clin Oncol 18(18): 3302–3317 10986064 10.1200/JCO.2000.18.18.3302

[bib5] Franic DM, Pathak DS (2003) Effect of including (versus excluding) fates worse than death on utility measurement. Int J Technol Assess Health Care 19(2): 347–361 12862192 10.1017/s026646230300031x

[bib6] Franic DM, Pathak DS, Gafni A (2005) Quality-adjusted life years was a poor predictor of women's willingness to pay in acute and chronic conditions: results of a survey. J Clin Epidemiol 58: 291–303 15718119 10.1016/j.jclinepi.2004.10.005

[bib7] Geels P, Eisenhauer E, Bezjak A, Zee B, Day A (2000) Palliative effect of chemotherapy: objective tumor response is associated with symptom improvement in patients with metastatic breast cancer. J Clin Oncol 18: 2395–2405 10856099 10.1200/JCO.2000.18.12.2395

[bib8] Gold M, Siegel JE, Russell LB, Weinstein MC (1996) Cost Effectiveness in Health and Medicine. New York: Oxford University Press

[bib9] Hillner BE, Smith TJ, Desch CE. (1992) Efficacy and cost-effectiveness of autologous bone marrow transplantation in metastatic breast cancer: estimates using decision analysis while awaiting clinical trial results. JAMA 267: 2055–2061 1552641

[bib10] Hopwood P, Watkins J, Ellis P, Smith I (2004) Clinical interpretation of quality of life outcomes: investigation in a randomized trial of gemcitabine plus paclitaxel compared to paclitaxel alone for metastatic breast cancer (MBC). Ann Oncol 15(Suppl 3); Abstract Book of the 29th ESMO Congress, Vienna, Austria, 29 October – 2 November 2004

[bib11] Jones SE, Erban J, Overmoyer B, Budd GT, Hutchins L, Lower E, Laufman L, Sundaram S, Urba WJ, Pritchard KI, Mennel R, Richards D, Olsen S, Meyers ML, Ravdin PM (2005) Randomized phase III study of docetaxel compared with paclitaxel in metastatic breast cancer. J Clin Oncol 23: 5542–5551 16110015 10.1200/JCO.2005.02.027

[bib12] Kind P, Dolan P, Gudex C, Williams A (1998) Variations in population health status: results from a United Kingdom national questionnaire survey. BMJ 316: 736–741 9529408 10.1136/bmj.316.7133.736 PMC28477

[bib13] Lam WT, Fielding R (2003) The evolving experience of illness for Chinese women with breast cancer: a qualitative study. Psycho-Oncology 12: 127–140 12619145 10.1002/pon.621

[bib14] Launois R, Reboul-Marty J, Henry B, Bonneterre J (1996) A cost-utility analysis of second-line chemotherapy in metastatic breast cancer: docetaxel versus paclitaxel versus vinorelbine. Pharmacoeconomics 10: 504–521 10169397 10.2165/00019053-199610050-00008

[bib15] Luoma ML, Hakamies-Blomqvist L (2004) The meaning of quality of life in patients being treated for advanced breast cancer: a qualitative study. Psycho-oncology 13(10): 729–739 15386642 10.1002/pon.788

[bib16] McCorkle MR (1973) Coping with physical symptoms in metastatic breast cancer. Am J Nurs 73(6): 1034–1038 4488330

[bib17] National Institute for Clinical Excellence (2004) Guide to the Methods of Technology Appraisal. London, UK: NICE 27905712

[bib18] Office of National Statistics (2001) National Statistics website: Population of the United Kingdom: by ethnic group http://www.statistics.gov.uk/cci/nugget_print.asp?ID=76

[bib19] Office of National Statistics (2003) National Statistics website, www.statistics.gov.uk

[bib20] O'Shaughnessy J, Miles D, Vukelja S, Moiseyenko V, Ayoub JP, Cervantes G, Fumoleau P, Jones S, Lui WY, Mauriac L, Twelves C, van Hazel G, Verma S, Leonard R (2002) Superior survival with capecitabine plus docetaxel combination therapy in anthracycline-pretreated patients with advanced breast cancer: phase III trial results. J Clin Oncol 20(12): 2812–2823 12065558 10.1200/JCO.2002.09.002

[bib21] Perez DJ, Williams SM, Christensen EA, McGee RO, Campbell AV (2001) A longitudinal study of health related quality of life and utility measures in patients with advanced breast cancer. Quality Life Res 10: 587–593 10.1023/a:1013193007095 11822792

[bib22] Rabin R, de Charro F (2001) EQ-5D: a measure of health status from the EuroQol Group. Ann Med 33: 337–343 11491192 10.3109/07853890109002087

[bib23] Sprangers MAG, Groenveld M, Arraras JI, Franklin J, te Velde A, Muller M, Franzini L, Williams A, de Haes HCJM, Hopwood P, Cull A, Aaronson NK (1996) The European Organization for Research and Treatment of Cancer breast cancer-specific quality-of-life questionnaire module: first results from a three-country field study. J Clin Oncol 14(10): 2756–2768 8874337 10.1200/JCO.1996.14.10.2756

[bib24] Torrance GW (1986) Measurement of health state utilities for economic appraisal. J Health Econ 5: 1–30 10311607 10.1016/0167-6296(86)90020-2

